# Efficacy of amoxicillin/clavulanic acid after surgical drainage of perianal abscess in the prevention of the development of anal fistula (PERIQxA study): study protocol for a multicenter randomized, double-blind clinical trial

**DOI:** 10.1186/s13063-024-07922-3

**Published:** 2024-02-15

**Authors:** Verónica Polaino Moreno, Antonio F. Caballero-Bermejo, Mariano Artés Caselles, Javier Serrano González, Xabier Remírez Arriaga, Natalia González Alcolea, Aritz Equisoain Azcona, Eva Iglesias García, José Luis Lucena de la Poza, Arsenio Sánchez Movilla, Belén Ruiz-Antorán, PERIQxA Study Group, Isabel Alonso Sebastián, Daniel Melero Montes, Carmen León Fernández, Jesús Álvarez Sánchez, Joaquín Muñoz Rodríguez, Juan González González, Miguel García-Oria Serrano, Ana Sánchez Ramos, Arturo García Pavía, Elena Jiménez Cubedo, Laura Román García de León, Maria Dolores Chaparro Cabezas, Javier López Monclús, Alberto Pueyo Rabanal, Matías Cea Soriano, Pedro Artuñedo Pe, Xiana Rial Justo, Miguel Suárez Sánchez, Lucía Gil Cidoncha, María del Pilar Martín Rodrigo, María Torguet Muñoz, Manuel Fernández Rodriguez, Cristián Grillo Marín, Celia Fidalgo Martínez, Javier Callau Pontaque, Pablo Galindo Jara, Laura Colao García, David Díaz Pérez, Ana Belén Gallardo Herrera, Enrique Esteban Agustí, Miguel A. Hernández Bartolomé, María Gutierrez Samaniego, César García Llorente, Lorenzo Rabadán Ruiz, Raquel Barriga Sánchez, Hector Ernesto Núñez Tasaico, Jaime Zabala Salinas, Natalia Almeida Romera, Raquel Ríos Blanco, Benito Miguel Josa Martínez, Eneida Bra Insa, Elena Sagarra Cebolla, Mónica García Aparicio, Paloma Garaulet González, Eduardo San Pío Carvajal, Miriam Fraile Vasallo, José Manuel Muros Bayo, Sergio Pedro Olivares Pizarro, Vicente Herrera Cabrera, Isaac Silvio Corrales Castillo, Elsa Pertejo Muñoz, Ricardo Ortega García, Ana Sánchez Gollarte, Rocío Maqueda González, Carlos Sáez Rodríguez, Mario Álvarez Gallego, Beatriz Díaz San Andrés, Ramón Cantero Cid, Carolina González Gómez, Inés Rubio Pérez, Fernando Tone Villanueva, Luis Asensio Gómez, Esteban Díaz Serrano, Blanca Monje Vera, Celia Castillo Marcos, Ana Belén Suárez Enriquez, Rebeca Abad Moret, Kriss Huerta Serracin

**Affiliations:** 1 General Surgery Department, Hospital Universitario Puerta de Hierro Majadahonda, Instituto de Investigación Sanitaria Puerta de Hierro Segovia de Arana, Majadahonda, Spain; 2 Clinical Pharmacology Department, Hospital Universitario Puerta de Hierro Majadahonda, Instituto de Investigación Sanitaria Puerta de Hierro Segovia de Arana, Majadahonda, Spain; 3 General Surgery Department, Hospital Universitario de Torrejón, Madrid, Spain; 4 General Surgery Department, Hospital Universitario Infanta Cristina, Madrid, Spain; 5 General Surgery Department, Hospital Universitario La Paz, Madrid, Spain

**Keywords:** Perianal, Fistula-in-ano, Abscess, Antibiotics, Quality of life, Colorectal surgery

## Abstract

**Background:**

Anorectal fistula, which is a relatively common pathology, is the chronic manifestation of the acute perirectal process that forms an anal abscess. The development of a fistula after incision and drainage of an anal abscess is seen in approximately 26–37%. Its treatment is a relevant topic, and the role of the use of antibiotic therapy in its prevention remains controversial, after the publication of several studies with contradictory results and several methodological limitations. Our hypothesis is that the combination of amoxicillin and clavulanic acid will reduce the incidence of anal fistula.

**Method:**

The aim of this study is to evaluate the efficacy of antibiotherapy after surgical drainage of perianal abscess in the development of perianal fistula. The PERIQxA study is a multicenter, randomized, double-blind controlled trial. The study has been designed to include 286 adult patients who will be randomly (1:1) assigned to either the experimental (amoxicillin/clavulanic acid 875/125 mg TDS for 7 days) or the control arm (placebo). The primary outcome measure is the percentage of patients that develop perianal fistula after surgery and during follow-up (6 months).

**Discussion:**

This clinical trial is designed to evaluate the efficacy and safety of amoxicillin/clavulanic in the prevention of perianal fistula. The results of this study are expected to contribute to stablish the potential role of antibiotherapy in the therapeutics for anal abscess.

**Trial registration:**

EudraCT Number: 2021–003376-14. Registered on November 26, 2021.

**Supplementary Information:**

The online version contains supplementary material available at 10.1186/s13063-024-07922-3.

## Background

An anorectal fistula (AF), also known as fistula-in-ano, is the chronic manifestation of the acute perirectal process that forms an anal abscess (AA). AA and AF should be considered the acute and chronic phase of the same anorectal disease [1]. It is a relatively common pathology and has an incidence of 3–4 and 1.04–8.6 per 100,000 cases every year in Spain and Europe respectively [2, 3]. Being an underdiagnosed pathology, its prevalence is probably even higher than estimated.

The cryptoglandular hypothesis states that AF develops from the blockage of anal glands and the perpetuation of chronic inflammation that predisposes fistula formation [4]. Between 26 and 37% of AF is diagnosed after surgical drainage of the abscess, most appearing in the first 3 months of postoperative follow-up [5, 6].

The development of fistula after drainage is associated with longer hospital stays, higher medical expenses, and patient discomfort [7]. AF manifests as an abnormal tract between the anorectal canal and the perianal skin and clearly affects patients’ quality of life [8]. Patients may experience rectal pain and pruritus, and the perianal skin may be excoriated and inflamed [9].

Perianal fistulous disease usually affects patients’ quality of life, especially in cases of long term or complex perianal fistulas [2, 10].

The treatment of abscess and fistula of cryptoglandular origin continues to be a relevant general surgery topic [11]. Historically, adjuvant antibiotic treatment has been limited to immunocompromised patients or those who presented with extensive cellulitis or sepsis [12]. The use of antibiotics in the development of AF after incision and drainage of an AA remains controversial. Published data are limited and sometimes contradictory. Different observational studies have been conducted with heterogeneous results [7, 13–15]. Only two randomized clinical trials have evaluated the prophylactic use of antibiotics in this setting [5, 6].

Considering the debate on the role of postoperative antibiotic therapy for patients with perianal abscesses and the importance of the prevention of anal fistulas, we have designed this study with the objective of determining the efficacy of antibiotherapy in preventing progression to perianal fistula, after surgical drainage of a perianal abscess.

Our hypothesis is that the combination of amoxicillin and clavulanic acid will reduce the incidence of anal fistula.

### Objectives

The aim of this trial is to evaluate the efficacy of antibiotherapy in the development of perianal fistula, after surgical drainage of perianal abscess.

### Study design

This is a phase 4, parallel group, randomized, double-blind, controlled, superiority, multicenter clinical trial. The protocol has been designed in accordance with the SPIRIT (Standard Protocol Items: Recommendations for Interventional Trials) guidelines (Supplementary Material).

Two hundred eighty-six patients (143 per arm) with perianal abscess requiring surgery will be enrolled from four different centers. Individuals fulfilling selection criteria will be randomized to receive amoxicillin/clavulanic acid or placebo at a ratio of 1:1.

## Methods: participants, interventions, and outcomes

### Participating centers

Study clinical sites included 4 public and private tertiary hospitals across different regions in Madrid (Spain). The only requirement for the participating study centers was to have a local research team with a general surgeon as principal investigator.

### Eligibility criteria

Inclusion criteria are as follows:

1. Male or female adult patients (18 or older)
2. First episode of perianal abscess
3. Oral feeding
4. Willingness and ability to comply with scheduled visits, treatment plan, laboratory tests, and other study procedures
5. Signed written informed consent in accordance with ICH/GCP and local legislation, obtained prior to any study procedure

Exclusion criteria are as follows:

- Allergy or intolerance to amoxicillin/clavulanic acid
- Contraindication to undergo any of the study procedures
- History of previous perianal surgery
- Any clinical condition, and/or analytical alteration that, *according to the investigator*, is considered significant enough to not participate in the study. The following are considered as such:
  - Previous perianal fistula or fistula diagnosed during surgery
  - Complex abscess
  - Marked cellulitis after surgical drainage
  - History of inflammatory bowel disease
  - History of anal cancer
  - Previous local trauma or radiotherapy
  - Diabetes mellitus
  - Immunosuppressed: oncology patients, chronic corticosteroid treatment or other immunosuppressants, chronic kidney disease
  - Prosthetic valves
  - Morbid obesity (BMI > 40)
  - Signs of severe sepsis
- Women with positive pregnancy test result or currently breastfeeding
- Unwillingness or inability to follow the procedures described in the protocol
- Inability to give written informed consent

### Informed consent

Investigators will obtain the subject’s informed consent in accordance with Spanish Law 14/2007 on Biomedical Investigation and the internationally ethical accepted guidelines. Eligible patients may only participate in the study after providing written informed consent approved by the research ethics committee. These forms are available from the corresponding author on request. Informed consent must be obtained before performing any specific study procedure. The process to obtain informed consent should be documented in the patient’s source documents (medical history).

## Study intervention

### Intervention description

Included patients who are randomized to the experimental group will receive amoxicillin/clavulanic acid, which will be administered orally at the doses and dosing regimen given in the Summary of Products Characteristics (SmPC): 875 mg/125 mg administered three times daily for 1 week.

Included patients who are randomized to the control group will receive PLACEBO tablets, to be administered three times daily for 1 week.

### Treatment assignment method

Randomization is 1:1 and will be performed using the eCRF.

### Masking

This is a double-blind study in which neither the patient nor the research team will know whether the patient has been randomized to the control or experimental group.

### Participant timelines

**Fig. 1:**
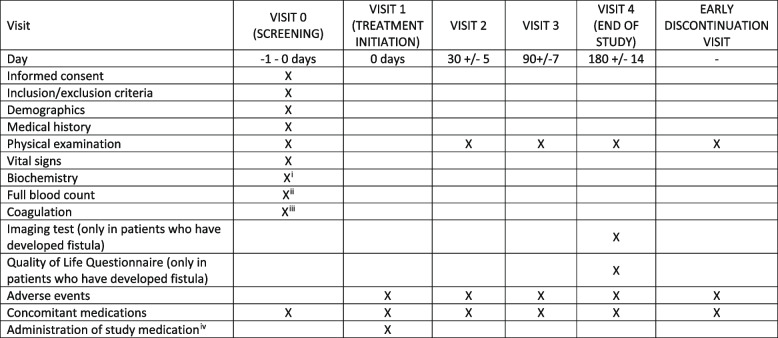
Schedule of visits and evaluations. “^i^” indicates the following: the biochemistry of the screening visit will be valid both on the day of the screening and in the 7 days prior to the screening. “^ii^” indicates the following: the FBC of the screening visit shall be valid both on the day of the screening and in the 7 days prior to the screening visit.“^iii^” indicates the following: the coagulation of the selection visit will be valid both on the day of the selection and in the 7 days prior to it. “^iv^” indicates the following: amoxicillin/clavulanic acid 875/125 mg is to be administered according to the SmPC: 1 tablet every 8 h for 7 days. Placebo should be administered under the same conditions: 1 tablet every 8 h for 7 days

The schedule of interventions and visits can be found in Fig. 1. All data obtained from these evaluations must be confirmed in the patient’s source documentation. All evaluations must be carried out within the time periods specified for each visit.

### Criteria for discontinuing or modifying allocated interventions

A patient may be removed from the study for the following reasons. However, whenever possible the patient should be followed regardless of their protocol adherence, as per the efficacy and safety evaluations:

- Patient withdraws consent or requests discontinuation from the study for any reason
- Termination of the study
- Lost to follow-up

Patients who withdraw from this study or are lost to follow-up after signing the informed consent form (ICF) will not be replaced. The reason for patient discontinuation from the study will be recorded on the appropriate case report form.

### Relevant concomitant care permitted or prohibited during the trial

There is no restriction regarding the use of medications and concomitant therapies. All medications (except study medication) and nondrug therapies administered during the study should be listed in the corresponding section of the concomitant medication case report form (CRF). The use of any concomitant treatment will follow the authorized conditions of use or the standard use in clinical practice.

### Provisions for post-trial care

There is no anticipated harm and compensation for trial participation. There is no provision for post-trial care. The follow-up of patients after their participation in the trial will be conducted according to the standard clinical practice protocols at each center.

## Outcomes

### Primary outcome measure

The primary outcome measure is the percentage of patients who develop perianal fistula after surgery and during follow-up (6 months).

### Secondary outcome measures

The secondary outcome measures include the following.

#### Evaluation of the effectiveness of the intervention

- Percentage of patients developing perianal fistula in the first 3 months after surgery
- Percentage of patients developing perianal fistula in the first month after surgery
- Time to onset of fistula (months)
- Complexity of the fistula according to the St James’s classification
- Presence of a new episode of perianal abscess during follow-up
- Need for scheduled surgery for perianal fistula treatment (number of interventions, time elapsed since initial event and technique used)
- Quality of life after the onset of perianal fistula

#### Safety and tolerability assessment

According to EMA recommendations (EMA/CPMP/EWP/280/96 Corr1), systemic safety shall be assessed on the basis of the adverse event record, the physical examination of patients, and the results of analytical studies.

### Sample size

To achieve 80% power to detect differences in the contrast of the null hypothesis *H*_0_:p1 = p2 using a bilateral chi-square test for two independent samples, taking into account that the significance level is 5%, and assuming that the proportion in the non-antibiotic group is 30%, the proportion in the antibiotic group is 15%, and that the proportion of experimental units in the reference group with respect to the total is 50%; it will be necessary to include 121 patients in the placebo group and 121 patients in the antibiotic group (242 patients in the study). Taking into account that the expected dropout rate is 15%, 143 patients per group would need to be enrolled, 286 patients in total.

### Data collection and management

All records will be collected in the eCRF, which will be completed by a trained and qualified investigator. Once the eCRF is completed, the original record will not be changed if any corrections are made. The completed eCRF will be reviewed by the clinical monitor.

Data entry and management will be guided by medical statistics experts. After reviewing and confirming that the established database is correct, the data will be locked by the main researchers and statistical analysts. The locked data or files will not be changed thereafter and will be submitted for statistical analysis by the research group. The Clinical Trial Unit of Instituto de Investigación Sanitaria Puerta de Hierro-Segovia de Arana, which does not have any competing interests, will be responsible for monitoring the data.

The study will be safely conducted in accordance with the protocol and applicable regulatory requirements under Good Clinical Practice, and data collection will be properly executed.

Analytical results necessary for the study will be routinely conducted in the clinical laboratories of participating centers following standard clinical practice.

### Statistical methods

The statistical analysis will be carried out following the principles specified in the International Conference on Harmonization Topic E9 (CPMP/ICH/363/96). The data will be analyzed using the SPSS statistical software (version 22.0; IBM Corp).

A detailed statistical analysis plan will be available before the database is declared closed. A summary of the general approach to statistical analysis is presented. No intermediate analyses are foreseen.

A descriptive analysis will be performed for all parameters overall and by arm at every study time-point. Categorical parameters will be presented by means of frequencies and percentages. Continuous parameters will be summarized by means of the appropriate descriptive statistics (mean ± standard deviation or median and interquartile range).

The efficacy and safety endpoints will be descriptively compared between study arms. Changes from baseline, when applicable, will also be summarized by study arm.

#### Primary endpoint

The primary endpoint is the of patients who develop perianal fistula after surgery and during follow-up (6 months). An estimate of the effect size will be obtained using the relative risk (RR) with its corresponding 95% confidence interval (95% CI). If an imbalance is observed between the baseline characteristics of the two groups, the outcome will be adjusted for these characteristics by binary logistic regression analysis. The dependent variable will be the occurrence of perianal fistula within 6 months; the main independent variable will be the group to which the patient has been randomized and, as covariates, those variables that are unbalanced. In this case, the odds ratio (OR) will be estimated together with its corresponding 95% CI. The significance level is set at 0.05.

An interim analysis is not planned as there is no anticipation of significant safety issues, and, given the multicenter nature of the study, rapid recruitment completion is expected. Nevertheless, if safety issues are detected, the sponsor reserves the right to prematurely interrupt the study.

### Plans to give access to the full protocol, participant-level data, and statistical code

These plans are not yet in place.

### Trial monitoring

The Clinical Trial Unit of Instituto de Investigación Sanitaria Puerta de Hierro-Segovia de Arana is responsible for project management, regulatory compliance, and trial monitoring.

The day-to-day support of the trial will be carried out by the principal investigator of the study, with the support of the members of the Clinical Trial Unit at the coordinating center, maintaining continuous contact with the principal investigators at each participating center.

### Adverse event reporting

Serious adverse events (SAEs) and grade 3 or 4 adverse events will be collected from the time of informed consent to day 29. SAEs will be followed up until the SAE has subsided, returned to baseline, or is stable.

### Plans for auditing trial conduct

Monitoring online visits will include, but not limited to, review of regulatory files, accountability records, CRFs, ICFs, medical and laboratory reports, site study intervention storage records, training records, and protocol and GCP compliance. On-site and off-site monitoring, central review of data collection, and remote source data verification will be allowed.

### Plans for communicating important protocol amendments to relevant parties (e.g., trial participants, ethical committees)

During the trial, any amendments to the protocol or consent materials will be approved by the REC before they are implemented.

### Dissemination plans

Following completion of the study, the results will be published in a scientific journal.

### Ethical and regulatory

The project, the final amended protocol (version 3; September 30, 2022), and the consent form have been reviewed and approved by the research ethics committee at Hospital Universitario Puerta de Hierro-Majadahonda (approval 17/21) and the Spanish Regulatory Authority (Spanish Agency of Medicines and Medical Devices).

This clinical trial has been registered on the European Clinical Trials Register (2021–003376-14).

## Discussion

Much controversy exists regarding the prophylactic use of antibiotics in the development of AF after incision and drainage of an AA. Its use is controversial and only weakly supported by current guidelines due to low-quality evidence [12]. To the best of our knowledge, there are only 2 clinical trials that have evaluated the use of prophylactic antibiotic therapy in this setting with in some cases inconclusive and contradictory results. Sözener et al. [6] concluded that antibiotic therapy with amoxicillin plus clavulanic acid had no protective effect regarding risk of fistula formation vs placebo at 1 year follow-up after surgical drainage of anorectal abscesses. This study could have had a negative result due to a low statistical power. On the other hand, Ghahramani et al. [5] concluded that postoperative prophylactic antibiotic therapy including ciprofloxacin and metronidazole play an important role in preventing fistula in-ano formation vs placebo at 3 months follow-up. In this study, a single dose of ceftriaxone was administered peri-surgically in both groups and 2 antibiotics other than the one we will assay in our study were used. As an additional difference, in this study only a 3-month follow-up was performed. A recent meta-analysis [16] on this issue concluded that further randomized trials are needed to fully clarify the role, duration, and type of antibiotics best suited for postoperative prevention of fistula following drainage of anorectal abscesses because the quality of evidence is low.

Thus, there is a substantial need to conduct appropriately powered randomized controlled studies in order to generate a reliable and conclusive evidence regarding the benefits and risks of these therapies. Our hypothesis is that the combination of amoxicillin and clavulanic acid will reduce the incidence of anal fistula.

## Trial status

Patient’s recruitment started on 12 April 2022 and is currently ongoing. It is anticipated that recruitment will be complete by the end of 2024.

### Supplementary Information

**Additional file 1.** 

## Data Availability

The datasets generated and/or analyzed during the current study will be made available. The corresponding authors will evaluate any request for data sharing and will consult with the steering committee after the publication of the main results. Requests can be sent to afcaballerobermejo@gmail.com.

## Abbreviations

AA: Anal abscess
AF: Anorectal fistula
BMI: Body mass index
CI: Confidence interval
CRF: Case report form
eCRF: Electronic case report form
EMA: European medicines agency
GCP: Good Clinical Practice
ICF: Informed consent form
ICH: International Conference of Harmonization
OR: Odds ratio
REC: Research ethics committee
RR: Relative risk
SAE: Serious adverse event
SmPC: Summary of Products Characteristics
